# Sex-dependent differences in type I IFN-induced natural killer cell activation

**DOI:** 10.3389/fimmu.2023.1277967

**Published:** 2023-12-15

**Authors:** Maria Pujantell, Nikolaos-Taxiarchis Skenteris, Janna Marieke Claussen, Benjamin Grünhagel, Rebecca-Jo Thiele, Marcus Altfeld

**Affiliations:** ^1^ Institute of Immunology, University Medical Center Hamburg Eppendorf (UKE), Hamburg, Germany; ^2^ Department Virus Immunology, Leibniz Institute of Virology (LIV), Hamburg, Germany

**Keywords:** natural killer cells, IFNα, type I IFNs, sex differences, antiviral, IFNα production, NK cell activation, plasmacytoid dendritic cells

## Abstract

Natural killer (NK) cells are important antiviral effector cells and also involved in tumor clearance. NK cells express IFNAR, rendering them responsive to Type I IFNs. To evaluate Type I IFN-mediated modulation of NK cell functions, individual Type I IFNs subtypes were assessed for their ability to activate NK cells. Different Type I IFN subtypes displayed a broad range in the capacity to induce and modulate NK cell activation and degranulation, measured by CD69 and CD107a expression in response to leukemia cell line K562. When including biological sex as a variable in the analysis, transwell co-cultures of NK cells with either male- or female-derived PBMCs or pDCs stimulated with the TLR7/8 agonist CL097 showed that NK cells were more activated by CL097-stimulated cells derived from females. These sex-specific differences were linked to higher CL097-induced IFNα production by pDCs derived from females, indicating an extrinsic sex-specific effect of Type I IFNs on NK cell function. Interestingly, in addition to the extrinsic effect, we also observed NK cell-intrinsic sex differences, as female NK cells displayed higher activation levels after IFNα-stimulation and after co-culture with CL097-stimulated pDCs, suggesting higher activation of IFNα-signaling transduction in female NK cells. Taken together, the results from these studies identify both extrinsic and intrinsic sex-specific differences in Type I IFN-dependent NK cell functions, contributing to a better understanding of sex-specific differences in innate immunity.

## 1 Introduction

The manifestations of many immune-mediated diseases differ between females and males, and in particular infectious and autoimmune diseases exhibit a strong sex bias (1–3). The mechanisms underlying these sex differences in diseases remain incompletely understood, but differences in the expression of genes located on the X chromosome, resulting from escape from inactivation of the second X chromosome (XCI) in female cells (4–8), and the immune-regulatory function of sex-hormones (9, 10) have been implicated. In particular, sex differences in IFNα production by pDCs after TLR7/8 stimulation have been described in several studies, and higher TLR7 expression due to *TLR7* gene escape from XCI and sex hormone-induced enhancement of TLR7 signaling have been shown to contribute to strengthen Type I IFN responses in females (11–16). Enhanced Type I IFN responses following TLR7-stimulation in females can mediate stronger antiviral immune responses in females (11–14), but also result in an enhanced risk for autoimmune diseases, such as Lupus Erythematosus (17). Furthermore, Type I IFNs have important effects on the development and function of other immune cells, including myeloid cells, T cells, B cells, and natural killer (NK) cells [reviewed here (18)].

NK cells are cytotoxic innate lymphocytes important for clearance of tumor and virus-infected cells (19, 20). NK cells are able to directly kill targeted cells via Perforin/GranzymeB expression and/or FastL, and produce cytokines that regulate the function of other immune cells. IFNα-stimulation is known to modulate NK cell cytotoxicity and effector mechanisms (21); therefore differences in IFNα production might lead to differences in NK cell effector function during viral infections. Sex and age have been reported to modulate frequencies and functions of NK cells (22–24), although some studies have led to discordant results (24–27). Recently, the X-linked epigenetic regulator UTX has been characterized in mice and shown to modulate NK cell numbers and effector function in a sex-dependent manner (28). UTX regulates chromatin accessibility (29), resulting in changes in the expression of genes involved in homeostasis and effector functions of NK cells (28, 30). To gain a better understanding of the NK cell-intrinsic and -extrinsic factors involved in sex-differential regulation of NK cell functions, we investigated the effects of Type I IFNs in modulating NK cell function in both males and females. The results from these studies show that NK cell-extrinsic differences in Type I IFN production between males and females and NK cell-intrinsic differences in the response to Type I IFN-stimulation can both contribute to sex-specific differences in NK cell function.

## 2 Materials and methods

### 2.1 Primary cell isolation and cell lines

Human peripheral blood mononuclear cells (PBMCs) were obtained from blood of healthy adult donors using a Ficoll-Plaque density gradient centrifugation Biocoll (Biochrom). Age of blood donors ranged between 18 and 35 years at the time of donation. PBMCs were used directly after isolation in transwell culture system. NK cells were isolated from PBMCs using negative selection kit EasySep human NK cell enrichment kit II (Catalog no. 19055, StemCell Technologies). Isolated NK cells were plated overnight before IFNα stimulation and degranulation assay; or used directly after isolation in transwell culture system. pDC were isolated from PBMCs using Plasmacytoid Dendritic Cell Isolation Kit II (Catalog no. 130-097-415, Miltenyi Biotec) and plated directly in transwell culture systems. All donors were recruited at the Leibniz Institute of Virology, Hamburg and provided written informed consent, studies were approved by the ethical committee of the Ärztekammer Hamburg (PV4780). Target cell line K562, a highly erythroleukemic cell line which does not express MHC class I molecules, was obtained from American Type Culture Collection (ATCC; Manassas, VA) and used for degranulation assays. K562 cell cultures were grown in RPMI-1640 supplemented with 10% heat-inactivated FBS and 1% Penicillin/Streptomycin (Sigma) at 37°C and 5% CO_2_. K562 cells were regularly tested for mycoplasma contamination internally.

### 2.2 Stimulation of primary cells

NK cells were cultured overnight (16h) with RPMI-1640 supplemented with 10% FBS, 1% Penicillin/Streptomycin (Sigma), 10 ng/ml IL-15 and 250 U/ml IL-2 (PeproTech) at a concentration of 2,5 × 10^5^ cells/ml. After, NK cells were pre-stimulated for 4h with either 10 IU/mL or 200 IU/mL of IFNα subtypes together with previously stated overnight medium culture conditions of IL-2 and IL-15. Recombinant human IFNβ (300-02BC, Preprotech); IFNα2 Human Interferon Alpha 2 (Alpha 2b) (11105–1), IFNα4 Human Interferon Alpha 4b (Alpha 4) (11180–1), IFNα7 Human Interferon Alpha J1 (Alpha 7) (11160–1), IFNα14 Human Interferon Alpha H2 (Alpha 14) (11145–1) were all provided by pbl assay science. IFN subtypes were resuspended according to the reported activity in human cells provided by the manufacturer. NK cells were then collected and used for degranulation assay and FACS staining.

Transwell co-culture consisted of 0,4 µm PET 24-well inserts (Thermo scientific/Sarstedt) in 24-well plates, which only allowed medium exchange and restricted NK cells to move to the lower chamber due to cell size restriction (data not shown). Transwell co-cultures were set by plating unstimulated or stimulated 1x10^6^ PBMCs or 4x10^4^ pDCs at the lower chamber, and 2x10^5^ isolated NK cells at the upper chamber for overnight (16h) stimulation with 1mL total volume per 24-well. PBMCs or pDCs were resuspended with TLR7/8 agonist CL097 (*In vivo*gen) with final concentration of 1µg/mL; positive control IFNα14 at 200U/mL; unstimulated condition with medium alone. After overnight co-culture, NK cells were collected and used for FACS staining; culture medium supernatant was collected and used for IFNα quantification.

### 2.3 NK cell degranulation assay

The frequency of degranulating NK cells was quantified by FACS staining, as previously described (31). Isolated NKs at 2,5x10^5^ cells/ml were stimulated with MHC devoid, K562 cells (ATCC), at an effector to target ratio of 1:5. Medium alone served as the negative control. Cells were stimulated with phorbol-12-myristate-13-acetate (PMA) (2.5 µg/ml) and Ionomycin (0.5 ug/ml) (Sigma) as a positive control. CD107a-BV510 antibody (BD Biosciences, San Jose, CA) was added directly to the tubes at 20 µl/ml. Cells were incubated for 1 h at 37°C in 5% CO_2_ after which Brefeldin A (Sigma) was added at a final concentration of 10 µg/mL, as well as, Monensin (Golgi-Stop 554724, BD Biosciences) at a final concentration of 6 µg/mL and incubated for an additional 4h at 37°C in 5% CO_2_ before FACS staining.

### 2.4 Characterization of NK cells using flow cytometry

Cells were stained for surface markers for 20 min at room temperature in PBS (Sigma). Extracellular antibodies used were as follows: LIVE/DEAD Fixable Near-IR (Thermo Fischer Scientific; L10119), Anti-CD3-BUV737 (BD Biosciences; 612750), Anti-CD14-BUV737 (BD Biosciences; 612763); Anti-CD14-BUV395 (BD Biosciences; 563561); Anti-CD19-BUV737 (BD Biosciences; 564303), Anti-CD19-BUV395 (BD Biosciences; 563549); Anti-CD56-PerCP-Cy5.5 (Biolegend; 304626), Anti-CD16-Pe-Cy7 (Biolegend; 302016), Anti-CD107a-BV510 (Biolegend; 328632), Anti-CD69-BV650 (Biolegend; 310934). Samples were then washed and fixed using 4% formaldehyde or BD Cytofix/Cytoperm Kit (BD biosciences) according to manufacturer’s directions. Flow cytometry analysis was performed on a LSRII instrument (BD Biosciences). A total of 50,000 to 250,000 events were acquired and analyzed using FlowJo software. The analysis was performed on gated cells that fell within the lymphocyte population, stained as live cells using Live/Dead stain. Cells were then gated for negative CD3/CD14/CD19 expression to exclude other cell populations, and NK cells were identified using CD56/CD16 gating strategy. Gating strategies are shown in **Supplementary Figure S1**. Within the NK cell population, we analyzed expression of CD107a and CD69 for each sample compared to their corresponding unstimulated control.

### 2.5 ELISA

Concentration of IFNα was measured in duplicates from cell culture supernatant after overnight stimulation of PBMCs or pDC cultured in transwell culture system using Human IFN Alpha Multi-Subtype ELISA Kit (Invitrogen; 41105-1) following manufacture’s recommendations. Supernatants were stored at -80°C. Positive controls and stimulated supernatants were diluted 1/100 for appropriate cytokine detection, as recommended.

### 2.6 Statistical analysis

Graphs were generated and statistical analysis performed using GraphPad Prism 9 (GraphPad Software). Wilcoxon matched pairs signed rank test was used for comparison between two paired groups. Mann-Whitney test was employed for comparison between two unpaired groups. Two-way ANOVA test was used for comparison between two independent groups. Spearman correlation was used to assess the relationship between two sets of measurements from the same sample.

## 3 Results

### 3.1 Different Type I IFN subtypes differentially modulate NK cell activation

**Figure 1:**
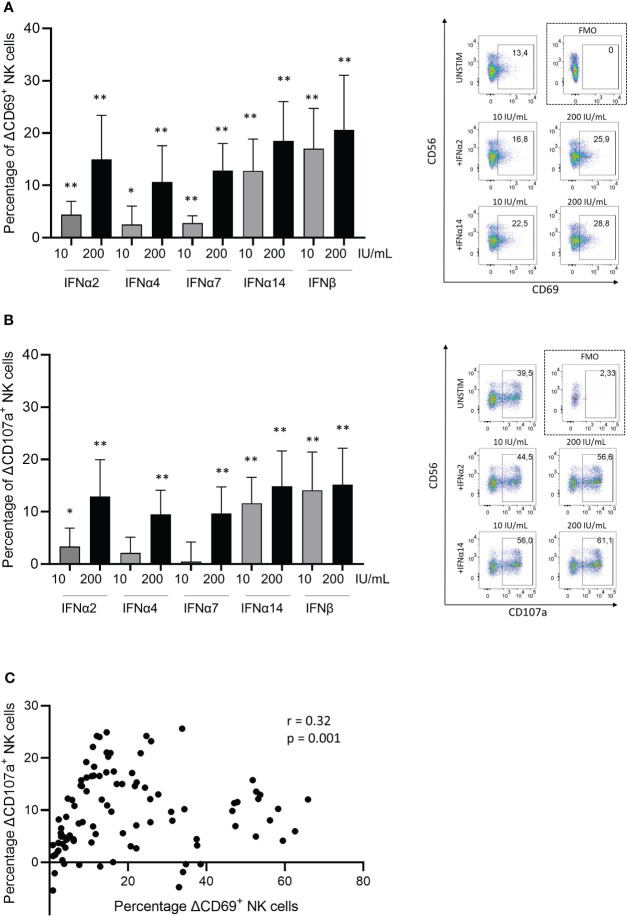
Type I IFN subtypes modulate natural killer cells *in-vitro*. Changes (presented as deltaΔ) in **(A)** activation measured by CD69+ expression on NK cells, and in **(B)** degranulation measured by CD107a+ expression on NK cells. Representative dot plots are shown on right side for CD69+ **(A)** and CD107a+ **(B)** expression on NK cells. PBMCs were stimulated with respective Type I IFN subtypes for 4h followed by a degranulation assay for 5h with K562 cell line. Plotted percentage delta values were calculated by subtracting the corresponding unstimulated condition with K562 cell co-cultures from each experiment. **(C)** Spearman correlation coefficient (r) between delta changes in activation (CD69) and delta changes in degranulation (CD107a) of NK cells after IFN-stimulation. Paired t test; non parametric Wilcoxon test; n=10. Spearman correlation; 100 XY pairs. *p<0.05; **p<0.005.

Previous studies have described that IFNα can induce NK cell function (21, 32) and that different IFNα subtypes mediate distinct antiviral immune functions (33–35). Here, we assessed the capacity of selected Type I IFN subtype proteins (IFNα2, IFNα4, IFNα7, IFNα14 and IFNβ) to induce activation and to promote degranulation of peripheral blood-derived NK cells by pre-incubating NK cells with different concentrations of IFN proteins prior to exposure to K562 cells. In line with previous studies using NK cells (36) and T cells (32, 37), different subtypes of Type I IFNs displayed different ability to induce NK cell activation, quantified by CD69 expression (**Figure 1A**), and degranulation, quantified by CD107a expression (**Figure 1B**). A representative example for CD69 expression (**Figure 1B**; right side) and CD107a expression (**Figure 1B**; right side) is illustrated; gating strategies are shown in **Supplementary Figure S1**. Significantly stronger activation and degranulation by NK cells were observed at higher IFN concentrations (200 U/mL) compared to low IFN concentrations (10 U/mL), demonstrating a dose-dependent activation of NK cells. While all Type I IFN subtypes induced significant NK cell activation at low and high levels, significant degranulation was not induced after IFNα4 and IFNα7 stimulation at low concentrations. Pre-incubation with IFNα14 and IFNβ induced the highest induction of NK cell activation and degranulation, already at low concentrations. Furthermore, increases in NK cell activation (CD69) and degranulation (CD107) following activation with Type I IFN subtypes significantly correlated (**Figure 1C**; r=0.32; p<0.001). Taken together, these data demonstrate that different Type I IFN subtypes can differentially enhance functional response of activated NK cells against target cells.

### 3.2 Extrinsic sex-differences from Type I IFN production impacts NK cell activation

**Figure 2:**
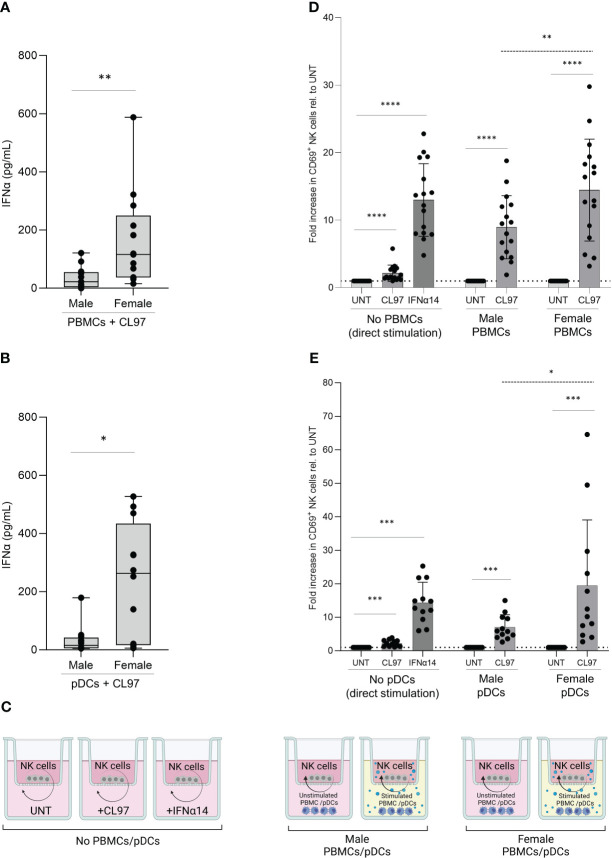
Sex differences in NK cell activation after TLR7/8 stimulation. **(A, B)** Poly-IFNα ELISA quantification of supernatants after overnight stimulation with 1ug/mL TLR7/8 agonist (CL097) of male and female-derived PBMCs **(A)** and pDCs **(B)**. Average of duplicates are represented in box and whisker plots, n=13 **(A)** and n=12 **(B)**. Mann-Whitney test. **(C)** Representation of transwell co-culture system. Overnight culture of NK cells, plated in upper chamber, with stimulated or unstimulated PBMCs or pDCs, plated in lower chamber. Image created with BioRender.com. **(D, E)** Relative activation levels measured by CD69+ NK cells normalized to their corresponding control condition (untreated). NK cells were cultured with control stimulation, as well as co-cultured with unstimulated or stimulated (CL097) PBMCs **(D)** or pDCs **(E)** from male and female donors in transwell system. Total of 16 independent donors in **(D)** or 12 independent donors in **(E)**, consisting of equal numbers of male and female donors. Absolute expression levels relative to untreated condition. Paired sets of a male- and female-donors were matched for each experiment including controls. Wilcoxon test paired (solid line); 2way ANOVA test (dotted line); *p<0.05**; p<0.005; ***p<0.0005; ****p<0.00005.

Sex-differences in the production of Type I IFNs by PBMCs after TLR7/8-stimulation have been shown in multiple studies (11), and consistent with these previous studies we observed significantly higher levels of IFNα protein in supernatants (median 5.3-fold higher) following stimulation of PBMCs derived from females with the TLR7/8 agonist CL097 compared to male-derived PBMCs after 16h (**Figure 2A**; p=0.005). The production of IFNα is primarily attributed to pDC subpopulation within PBMCs (38). We therefore also stimulated isolated pDCs with the TLR7/8 agonist CL097, and observed comparable ranges of IFNα-production in supernatants (**Figure 2B**). As observed for PBMCs, female-derived pDCs produced significantly higher levels of IFNα (median 17-fold higher; p=0.008) than male-derived pDCs (**Figure 2B**). Using the transwell co-culture system depicted in **Figure 2C**, we subsequently aimed to determine the effects of higher production of IFNs after TLR7/8 stimulation by female PBMCs/pDCs on NK cell functions. Using NK cells derived from the same donor, we compared the effect of male- versus female-derived PBMCs, either non-stimulated or stimulated with CL097. Significantly higher NK cell activation levels, quantified by CD69 expression, were observed after NK cell co-incubation with CL097-stimulated PBMCs derived from females compared to males (**Figure 2D**; p=0.03 dotted line), showing that higher IFNα production from stimulated female-derived PBMCs can trigger higher NK cell activation. These findings were replicated using CL097-stimulated pDCs (**Figure 2E**; p=0.02 dotted line), thus demonstrating that pDCs are the primary source of IFNα production after TLR7/8-stimulation regulating NK cell activation (**Figure 2E**). Further comparison of CD69 expression between CD56^bright^ and CD56^dim^ NK cells subset showed that CD56^dim^ NK cells exhibit the highest fold change in response to activation (**Supplementary Figures S1B–D**), in line with previous studies (23). Taken together, female-derived pDCs from PBMCs are able to induce stronger NK cell activation compared to male-derived pDCs after TLR7/8 stimulation, demonstrating an extrinsic sex-specific effect resulting from higher IFNα production of female pDCs that can modulate NK cell functions.

### 3.3 Sex-bias in Type I IFN production unmasks intrinsic sex-differences in NK cell activation

**Figure 3:**
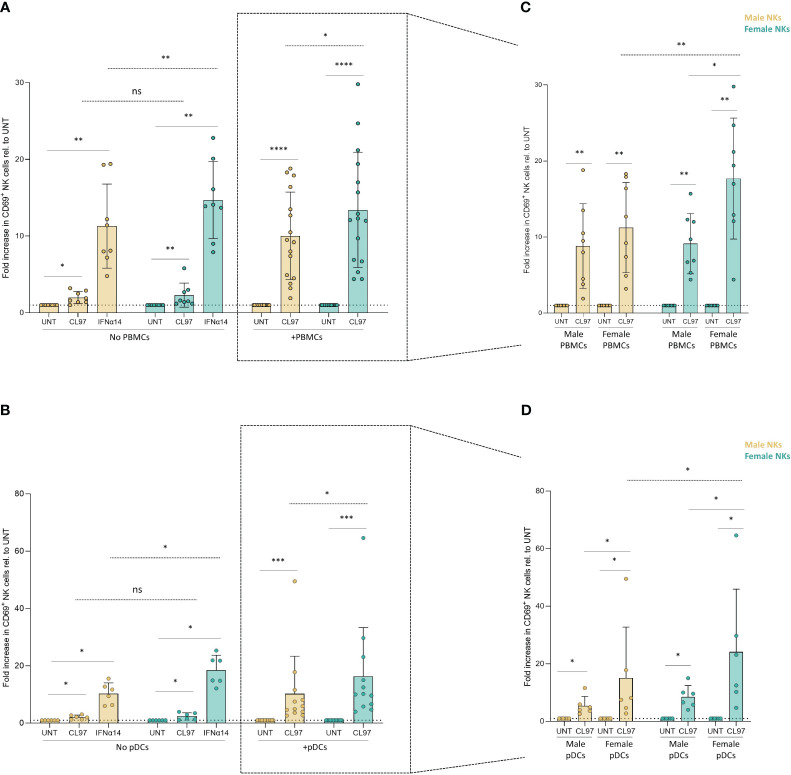
NK cell-intrinsic sex differences in response to TLR7/8 stimulation. **(A, B)** Relative activation of CD69+ NK cells, separated by sex, after overnight cultures with control stimulations, as well as co-cultures with unstimulated and CL097-stimulated PBMCs **(A)** or pDCs **(B)** in transwell system. Activation levels were relativized to their own control condition (untreated). **(C, D)** Relative activation levels measured by CD69+ NK cells by further separating the sex of NK cells- and PBMCs-derived donors **(C)** or pDCs-derived donors **(D)**. Activation levels were relativized to their own control condition (untreated). Total of 16 independent donors in **(D)** or 12 independent donors in **(E)**, consisting of equal number of male and female donors. Paired sets of a male- and female-donors were matched for each experiment, including controls. Male NK cells (yellow); female NK cells (turquoise). Wilcoxon test paired (solid line); 2way ANOVA test (dotted line); ns, non-significant; *p<0.05; **p<0.005; ***p<0.0005; ****p<0.00005.

Based on the recent description that sex-differences in UTX expression can result in stronger NK cell function in female mice (28), we aimed to investigate potential intrinsic sex-differences in the response of NK cells to Type I IFNs. Direct exposure to IFNα14 resulted in significantly enhanced activation of both male- and female-derived NK cells compared to their untreated condition (**Figure 3A**, no PBMCs and **Figure 3B**, no pDCs). However, IFNα14-induced activation was higher in NK cells derived from females (15-fold increase) compared to NK cells derived from males (11-fold increase) (**Figure 3A**, no PBMCs; p=0.005 dotted line), and a comparable stronger activation of NK cells derived from females (18- versus 10-fold increase; p=0.01) was observed after IFNα14-induced activation in **Figure 3B** (No pDCs; dotted line). We next used the above described transwell co-culture systems of NK cells with CL097-stimulated PBMCs, or CL097-stimulated pDCs, to investigate the combined effects of sex differences in Type I IFN production and in IFN-responsiveness of NK cells. Female-derived NK cells exhibited significantly enhanced activation compared to male-derived NK cells in co-culture with CL097-stimulated PBMCs (**Figure 3A**, +PBMCs; p=0.01 dotted line within rectangle) or pDCs (**Figure 3B**, +pDCs; p=0.008 dotted line within rectangle), independent of the sex of the PBMC/pDC donor. A subsequent integrated analysis combining both the sex of the NK cell donors and the PBMC/pDC donors as variables showed that strongest activation of NK cell was observed when CL097-stimulated PBMCs (**Figure 3C**; p=0.004 dotted line) or pDCs (**Figure 3D**; p=0.03 dotted line) were derived from females and incubated with NK cells derived from females. Taken together, these results demonstrate that NK cells derived from females are more strongly responding to Type I IFN-mediated activation than NK cells derived from males, and that this effect is strongest when co-incubated with IFNα-producing cells derived from females.

## 4 Discussion

Sex represents an important biological variable in immunology, and recent data suggest that NK cell frequencies and function are shaped by sex-specific factors (28, 39). In the current study, we identified both NK cell–extrinsic and -intrinsic factors that resulted in sex-specific differences in NK cell activation in response to Type I IFNs, leading to stronger NK cell activation in females.

NK cells play a central role in antiviral immune responses, and are modulated by signaling molecules such as Type I IFNs released by innate cells as first line of defense after viral sensing. We observed significant *in vitro* activation of NK cells in response to different IFNα subtypes, with the strongest activation induced by IFNα14 and IFNβ subtypes. The observed effects of different Type I IFN subtypes on NK cell activation and degranulation followed a similar pattern as the reported binding affinities of the tested IFNα subtype to IFNAR2, and to a lesser extend to IFNAR1 (33, 40, 41). While the precise mechanisms underlying these differential effects of different IFNs are not completely understood, binding of IFNs to both IFNAR1 and IFNAR2 is required to initiate a signaling response, and differences in binding affinities to the receptor subunits IFNAR1 and IFNAR2 have been suggested to allow the fine-tuning of different pleiotropic effects of Type I IFNs (41). Specific effects of IFNα subtypes on antiviral immune functions have furthermore been shown to be dependent on the investigated cell types and/or viral pathogens (33, 42) [reviewed here (43)]. The strong activity of IFNα14 and IFNβ subtypes on NK cell activation and degranulation, and low activity of IFNα4, observed in our studies, are in line with recently published data on the effects of Type I IFN subtypes on NK cells (36). Moreover, IFNα14 has been shown to be one of the strongest inducers of ISG transcription (44) and reported to protect hepatocytes from HBV infection (45). Taken together, these data demonstrate the critical and differential role that Type I IFNs play in the regulation of NK cell functions.

While sex differences in the production of Type I IFNs upon TLR7 stimulation by plasmacytoid dendritic cells (pDC), the major producers of Type I IFNs in the peripheral blood, have been well established (5, 11, 46, 47), the downstream implications of these sex differences in Type I IFN production on immune cell functions are not well understood. We therefore specifically investigated the functional consequences of sex differences in Type I IFN production by PBMCs for NK cells, using a transwell co-culture system, and validated that the observed effects from Type I IFN production originated from the stimulated pDCs subset within peripheral blood mononuclear cells (PBMCs). As expected from previous studies (5, 11, 46, 47), we observed that female-derived PBMCs exhibit stronger Type I IFN production compared to male-derived PBMCs upon TLR7 stimulation, in line with the sex-dependent effects reported for TLR7-stimulated pDCs (5, 12, 13, 46). These sex differences in Type I IFN production resulted in functional differences, as stimulated female-derived cells induced higher NK cell activation. Overall, these results show higher NK cell activation levels after co-incubation with female TLR7-stimulated PBMC and/or pDCs, resulting from stronger IFNα production. Further functional characterization of NK cells, using activation assays of overnight IFN-stimulated NK cells against K562 cells, did not display significant sex differences in CD107a, IFNγ, or TNFα expression levels (data not shown), possibly due to partial exhaustion or functional impairment of NK cells following prior overnight activation. While we cannot exclude a potential sex-specific effect of other cytokines induced by TLR7-stimulation, multiplex cytokine analysis of additional inflammatory and interferon-stimulated cytokines showed no significant differences between TLR7-stimulated cultured medium collected from male and female PBMCs (data not shown). Taken together, the *in-vitro* co-culture system provided a valuable tool to study sex-dependent effects of Type I IFN production on NK cells, and revealed a NK cell-extrinsic mechanism for stronger NK cell responses in females compared to males.

Recently, sex-dependent differences in UTX expression, which is located on the X chromosome, have been identified as a NK cell-intrinsic factor modulating accessibility to chromatin and enabling more rapid responses of female NK cells to viral infections (28). To investigate NK cell-intrinsic differences in responses to IFNs between males and females, we next compared the responses of NK cells derived from female and male donors using the transwell co-culture system. Female-derived NK cells showed significantly enhanced NK cell activation compared to male-derived NK cells exposed either directly to IFNα14 or to CL097-stimulated PBMCs and pDCs. Importantly, the activation of female-derived NK cells was strongest when co-incubated with PBMCs or pDCs from females, demonstrating a synergistic effect of NK cell-intrinsic and -extrinsic factors that enhanced NK cell responses in females. Further studies are needed to investigate the underlying mechanisms of these sex-specific differences leading to higher activation rates, including the previously described effect of the UTX gene encoded by the X chromosome on murine NK cells (28), or potential effects of other transcription factors that are differentially expressed between males and females, such as IRF5 (15). Taken together, these studies show that both, NK cell-extrinsic and –intrinsic differences in response to Type I IFNs can mediate sex-specific differences in NK cell responses, contributing to our understanding of differences in innate immune responses between males and females.

## Author’s note

We would like to clarify that terminology used in this manuscript, specifically the terms “female NK cells” and “male NK cells”, is intended to simplify the biological implications of the influence of sex hormones and the presence of sex chromosomes during life in these cells. We want to clarify that sex and gender are two separate factors. This manuscript only considered the designated sex recorded by the Healthy Cohort Hamburg (HCHH) to the best of our knowledge. However, we would like to emphasize the importance of using respectful terminology and be mindful regarding the diversity of human individuals and their personal identification.

## Data availability statement

The raw data supporting the conclusions of this article will be made available by the authors, without undue reservation.

## Ethics statement

The studies involving humans were approved by Ethical committee of the Ärztekammer Hamburg (PV4780). The studies were conducted in accordance with the local legislation and institutional requirements. The participants provided their written informed consent to participate in this study.

## Author contributions

MP: Conceptualization, Writing – review & editing, Data curation, Formal Analysis, Investigation, Visualization, Writing – original draft, Methodology. N-TS: Investigation, Methodology, Visualization, Writing – review & editing. JC: Investigation, Writing – review & editing. BG: Investigation, Writing – review & editing. R-JT: Investigation, Writing – review & editing. MA: Conceptualization, Funding acquisition, Resources, Supervision, Writing – review & editing, Project administration.

## Supplementary material

Supplementary Figure 1 Gating strategy for identifying NK cells and activation levels in CD56^bright^ and CD56^dim^ NK cells subsets. **(A)** Representative gating strategy to identify NK cells from PBMCs. NK cells were gated as singles by using SSC-H and FSC-H; negatively selected with LiveDead-, CD3-, CD14-, CD19- staining; and further gated by CD56 and CD16. **(B)** Representative zebra plots for a male-derived NK cell donor (left side) and female-derived NK cell donor (right side) for all conditions tested. Activation levels, measured by CD69, for the evaluation of CD56^bright^ and CD56^dim^ NK cells. (C and D) Relative activation levels measured by CD69+ CD56^bright^ **(C)** or CD56^dim^ **(D)** NK cells normalized to their corresponding control condition (untreated). NK cells were cultured with control stimulation, as well as co-cultured with unstimulated or stimulated (CL097) PBMCs from male and female donors in transwell system. Total of 16 independent donors, consisting of 8 male and 8 female donors. Activation levels were relativized to their own control condition (untreated). Wilcoxon test paired (solid line); 2way ANOVA test (dotted line); *p<0.05; **p<0.005.
