# Supplementary figures and images for: Sex-dependent differences in type I IFN-induced natural killer cell activation
Source: Front Immunol. 2023 Dec 15;14:1277967. doi: 10.3389/fimmu.2023.1277967 (PMC10757368; doi:10.3389/fimmu.2023.1277967)

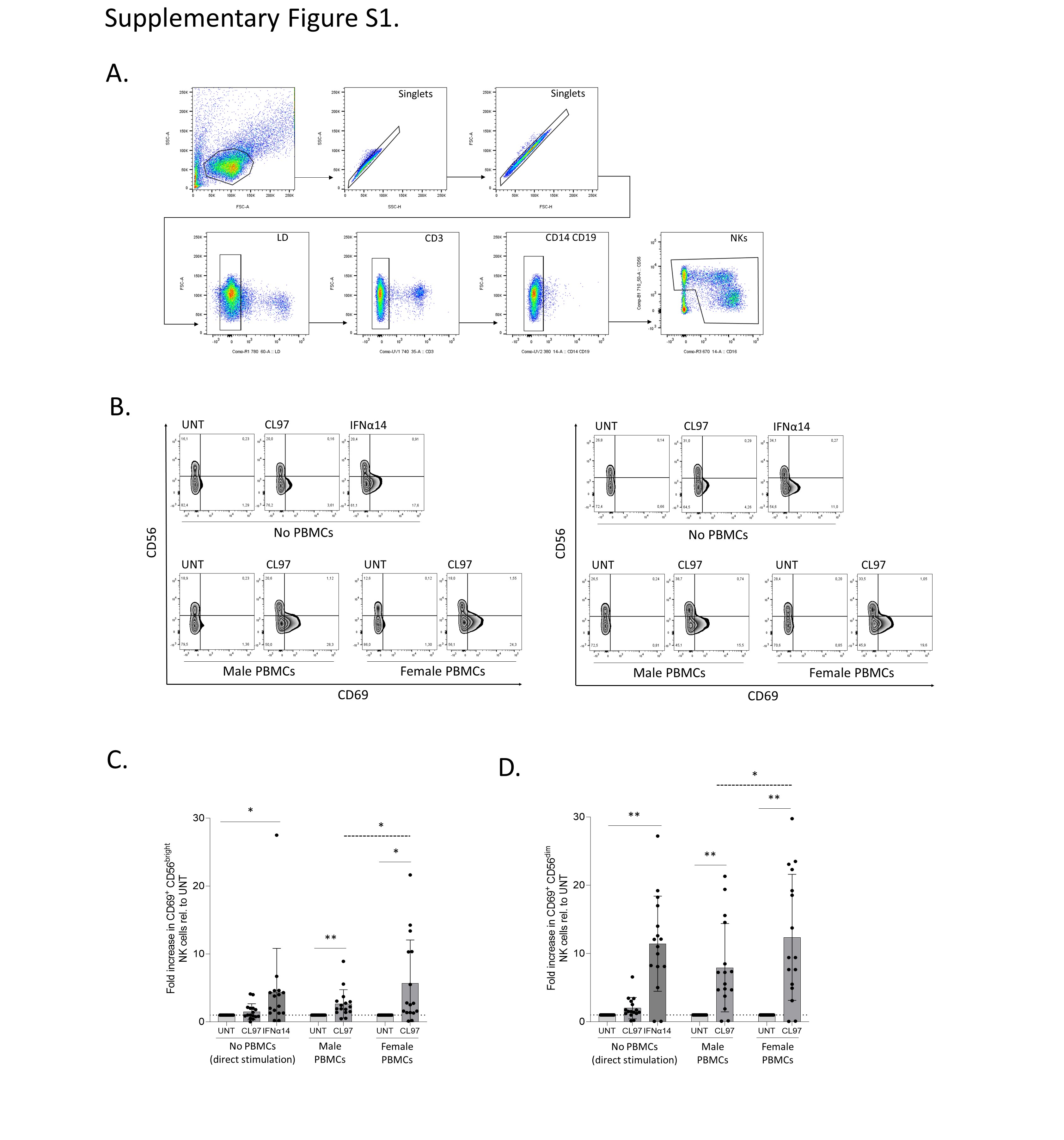

Supplement: Supplementary Figure 1 — Gating strategy for identifying NK cells and activation levels in CD56bright and CD56dim NK cells subsets. (A) Representative gating strategy to identify NK cells from PBMCs. NK cells were gated as singles by using SSC-H and FSC-H; negatively selected with LiveDead-, CD3-, CD14-, CD19- staining; and further gated by CD56 and CD16. (B) Representative zebra plots for a male-derived NK cell donor (left side) and female-derived NK cell donor (right side) for all conditions tested. Activation levels, measured by CD69, for the evaluation of CD56bright and CD56dim NK cells. (C and D) Relative activation levels measured by CD69+ CD56bright (C) or CD56dim (D) NK cells normalized to their corresponding control condition (untreated). NK cells were cultured with control stimulation, as well as co-cultured with unstimulated or stimulated (CL097) PBMCs from male and female donors in transwell system. Total of 16 independent donors, consisting of 8 male and 8 female donors. Activation levels were relativized to their own control condition (untreated). Wilcoxon test paired (solid line); 2way ANOVA test (dotted line); *p<0.05; **p<0.005. [file Image_1.jpeg]
